# Growth responses and physiological and biochemical changes in five ornamental plants grown in urban lead‐contaminated soils

**DOI:** 10.1002/pei3.10013

**Published:** 2020-05-21

**Authors:** Xiliang Song, Chenxiang Zhang, Weifeng Chen, Yihao Zhu, Yueying Wang

**Affiliations:** ^1^ College of Resources and Environment Shandong Agricultural University Tai’an China; ^2^ Shandong Provincial Engineering & Technology Research Center for Phyto‐microremediation in Saline‐alkali Land Shandong China; ^3^ College of Biological Sciences and Technology Beijing Forestry University Beijing China

**Keywords:** comprehensive evaluation value, ornamental plants, phytoremediation, soil lead, stress tolerance

## Abstract

An increasing concentration of lead (Pb) in urban contaminated soil due to anthropogenic activities has been a global issue threatening human health. The use of urban ornamental plants as phytoremediation of Pb‐contaminated soil is a new choice. In the present experiment, the physiological and biochemical response of five ornamental plants to increase in concentrations of C_4_H_6_O_4_Pb·H_2_O in the soil were measured to investigate these plans’ Pb tolerance strategies and abilities. Our results showed that Pb stress significantly inhibited the growth and the biomass of all the plants. The root activity (RA), net photosynthetic rate (*P*_n_), and chlorophyll (Chl) content in Pb‐stressed leaves were significantly decreased, whereas the leaf proline (Pro), soluble sugar (SS), and membrane stability index (MSI) were remarkable increased compared with those in the control group. By application of all‐subsets regression and linear regression, the reduction in photosynthetic capacity in the five plants is mainly due to the decrease in the leaf Chl content caused by Pb stress. The bioconcentration factor (BCF) in *Canna generalis* was greater than 1, while in the other plants were lower than 1, suggesting that *Canna generalis* had the highest Pb accumulation ability. The translocation factor (TF) in all the plants were lower than 1, suggesting that Pb preferentially accumulated in the external part of roots. By calculating the comprehensive evaluation value (CEV), *Iris germanica* L. was found to be the most sensitive species, and *Canna generalis* was the most tolerant species, to Pb stress among the five ornamental plants.

## Graphical abstract

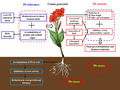

## 1 INTRODUCTION

Heavy metal pollutants released by series of anthropogenic activities including metalliferous mining, use of chemical fertilizers with heavy metal impurities, sewage disposal, municipal landfills, road traffic, and agricultural activities, have become a global environmental problem (Gichner, Žnidar, & Száková, 2008; Kang et al., 2015; Khuzestani & Souri, 2013; Laidlaw, Zahran, Mielke, Taylor, & Filippelli, 2012; Ray & George, 2010; Sarma, Islam, & Prasad, 2017; Verma, Das, & Kumar, 2017). Unlike other pollutants, the heavy metals are not biodegradable and can remain in soil and water at high concentrations for hundreds of years (Verma et al., 2017), ultimately resulting in serious damage to the biotic communities (Auger et al., 2013; Sarma et al., 2017).

Lead (Pb), which is highly phytotoxic, has been recognized as one of the most abundant heavy metals worldwide, and its accumulation is regarded as irreversible and highly toxic to plants (Qin et al., 2018; Shahid, Pinelli, Pourrut, Silvestre, & Dumat, 2011). Although Pb is not an essential element for plants, it is found to have strong affinity to organic and/or inorganic ligands in the soil (Cunningham, Berti, & Huang, 1995; Rodríguez‐Seijo, Andrade, & Vega, 2017), and it can be easily absorbed by plant roots and accumulated in plant tissues even at a low level (Chandrasekhar & Ray, 2019; Johnson, 1998). The hazardous effects of Pb on plant morphological, metabolic, physiological, and biochemical processes have been widely studied (Rucinska, Sobkowiak, & Gwozdz, 2004; Strubińska & Hanaka, 2011; Verma & Dubey, 2003; Yongsheng, Qihui, & Qian, 2011). Studies have shown that excessive amounts of Pb in soil produce excessive reactive oxygen species (ROS; Reddy, Kumar, Jyothsnakumari, Thimmanaik, & Sudhakar, 2005), increase in catalytic enzyme activities (such as polyphenol oxidase, shikimate dehydrogenase and phenylalanine ammonialyase; Wang et al., 2011) and membrane permeability (Sharma & Dubey, 2005), induce leaf chlorosis (Chettri, Cook, Vardaka, Sawidis, & Lanaras, 1998) and cell organelle degeneration (Gratão, Polle, Lea, & Azevedo, 2005), block root elongation (Kopittke, Asher, Blamey, & Menzies, 2007; Liu, Reid, & Smith, 2000), delay seed germination (Lamhamdi, Bakrim, Aarab, Lafont, & Sayah, 2011), disturb the uptake and translocation of nutrient elements (Gopal & Rizvi, 2008), and inhibit plant photosynthesis and respiration (Islam et al., 2008; Rucińska, Waplak, & Gwóźdź, 1999), resulting in a considerable reduction of both vegetative and reproductive plant growth (Hadi, Bano, & Fuller, 2010; Johnson & Eaton, 1980; Sharma & Dubey, 2005; Yongsheng et al., 2011).

The non‐biodegradable and immobile features of Pb due to its strong bond formation with soil organic and/or inorganic ligands makes its removal from contaminated soils very difficult (Cunningham et al., 1995; Dávila et al., 2011a,2011b). Until now, many physical, chemical, and biological technologies have been developed to remediate metalliferous sites. However, only a few technologies are both economical and cost effective. Among those remediation methods, phytoremediation is an eco‐friendly and successful approach for the remediation of toxic metals in polluted soils (Chandrasekhar & Ray, 2019; Cunningham & Berti, 1993; Noufal, Maalla, Noufal, & Hossean, 2017; Salazar & Pignata, 2014). It is a plant‐based technology that utilizes specific hyper‐accumulator plants to retain, remove or reduce toxic metals and metalloids in soil. However, this technology will fail if the concentration of available metal in the soil exceeds its permissible limits (Muszynska, Hanus‐Fajerska, & Ciarkowska, 2018). Hence, the identification of native plant species with metal tolerance, high metal exaction capacity and high biomass yield is essential for the phytoremediation of heavy metal‐contaminated soils (Asgari, Ghorbanpour, & Nikabadi, 2017; Chandrasekhar & Ray, 2017; Gerhardt, Gerwing, & Greenberg, 2017; Macek, Macková, & Káš, 2000).

Urban soil differs from natural soil, because anthropogenic activities can intensely affect its characteristics, and human activities account for 90% of environmental pollution (Gąsiorek, Kowalska, Mazurek, & Pająk, 2017; Puskás & Farsang, 2009). In China, for example, it has been reported that the average concentrations of As, Cd, Cr, Cu, Hg, Ni, Pb, and Zn in the 31 Chinese provincial capital cities reached 11.03, 0.25, 67.92, 32.80, 0.21, 27.23, 36.28, and 99.21 mg/kg, respectively (Zhang, Zha, Guo, Meng, & Zhou, 2018), posing a serious threat to public health. Ornamental plants planted on urban roadsides have great potential for phytoremediation because the absorbed Pb by plant roots cannot enter the food chain to cause Pb toxicity to humans. Thus, identifying native ornamental plants that are able to survive in severe Pb contaminated soil and that show robust Pb accumulation is a new approach for the effective remediation of urban Pb‐contaminated soils. In this study, physiological and biochemical parameters of five Pb‐stressed ornamental plants (*Hemerocallis fulva*, *Iris germanica* L., *Canna generalis*, *Pennisetum clandestinum*, and *Miscanthus sinensis*), which have been widely used for urban greening in China, were measured and analyzed to investigate and compare their Pb tolerance strategies and abilities. Overall, the major objective of the present experimental study was (a) to delineate the growth responses and physiological and biochemical changes in five ornamental plants in reponse to increasing levels of Pb in the soil within a six‐week period; (b) to assess the Pb accumulation potential of the five different plants, and (c) to explore the physicochemical mechanisms of plants with tolerance to extensive Pb stress.

## 2 MATERIALS AND METHODS

### 2.1 Plant material and growth conditions

The experiment was conducted at Shandong Agriculture University (36°09′N, 117°09′E, 128 m above sea level) in Taian, Shandong Province, China. Healthy and uniformity seeds of *Hemerocallis fulva*, *Iris germanica* L., *Canna generalis*, *Pennisetum clandestinum*, and *Miscanthus sinensis* were purchased from a local market. All seeds were soaked in distilled water to improve the germination percentage. After soaking in distilled water for at least 24 hr, the seeds were placed in germinating beds consisting of 5 cm diameter Petri dishes, filled with disinfected peat. Each Petri dish contained one seed. The dishes were incubated at 25°C and 75% relative humidity in an artificial climate chamber (RXZ‐380, made by Ningbo Southeast Instrument Company), with light period and intensity of 14 hr/day and 1,200 mol m^−2^ day^−1^, respectively. After three weeks of germination, each individual seedling with 4–5 leaves was transplanted into a plastic plot (18 cm in diameter and 22 cm in depth) which was filled with 5.5 kg Pb‐treated soils. The soil in each plot was watered with deionized water to 60% soil moisture.

The soil samples were collected from the local surface soil (0–20 cm). After air drying inside the laboratory for two weeks, the soil samples were sieved through a 2‐mm mesh. The soil filled pots were kept for 5 days for maturation followed by planting. The soil was classified as Alfisols under an Ultisol in USDA taxonomy and the soil pH was 7.5, the organic matter content 10.5 g/kg, the total nitrogen 0.65 g/kg, available phosphorus content 0.032 g/kg, available potassium content 0.032 g/kg and available Pb concentration was 9.85 mg/kg.

### 2.2 Experimental design

Six treatments (0, 50, 100, 200, 500, and 1,000 Pb mg/kg) with three replicates were set up and designated as Pb0, Pb50, Pb100, Pb200, Pb500, and Pb1000 in the experiment, respectively. Altogether 90 individual pots were maintained in the study. Each of the treatment levels was prepared by dissolving the respective concentrations of Pb equivalent to C_4_H_6_O_4_Pb·H_2_O in 500 ml of distilled water. The soil in each plot was spiked with the required levels of Pb solutions. Before transferring the plant seedling to the plot, the Pb‐spiked soil was left for two weeks to reach equilibration.

### 2.3 Determination of chlorophyll (Chl) content

Chl *a*, *b* and total Chl contents in fresh leaves were extracted by acetone and measured according to the method described by Hiscox and Israelstam (1979).

### 2.4 Determination of proline (Pro)

The extraction procedure and colorimetric determination of Pro in plant cells was carried out according to Bates, Waldren, and Teare (1973). Briefly, 1.0 g leaf sample was homogenized in 3% (w/v) aqueous sulfosalicylic acid. The homogenate was then filtered through two layers of filter paper to obtain a clear filtrate. After addition of the glacial acetic acid and acid ninhydrin mixture to 1 ml of the filtrate, the reaction mixture was heated in a in 100°C water bath for 1.0 hr. The reaction was terminated in an ice bath. The absorbance was read at a wavelength of 546 nm. The Pro concentration was calculated using a standard curve.

### 2.5 Determination of soluble sugars (SS)

The SS in leaves were extracted and identified following the method of Nelson (1944). Briefly, 0.5 g of fresh leaves were ground in 80% neutral aqueous ethanol and heated in a 100°C water bath for 10 min. The extract was centrifuged at 5,000 rpm for 10 min, and 1.0 ml supernatant was added to 4 ml anthrone reagent followed by heating in a 100°C water bath for 10 min. The reaction was stopped by incubation in a water bath at room temperature (20°C) for 5 min. The absorbance was measured at 630 nm.

### 2.6 Measurements of the leaf net photosynthetic rate (*P*_n_)

Healthy and fully expanded leaves in three plants from each Pb treatment from different pots were chosen to measure the leaf *P*_n_ using a portable photosynthesis system (LI‐6400, Li‐COR Inc.) with a red–blue LED light source as the illumination. The measurements were conducted from 9:00 to 11:00 a.m. on a sunny day.

### 2.7 Membrane thermostability index (MTI)

The MTI was determined in 10 leaf discs from fully expanded young leaves by measuring electrolyte leakage according to the method of Sullivan and Ross (1979). After washing in deionized water, 20 ml deionized water was added to the leaf discs in a capped tube and incubated at 25°C for 24 hr. The values of electrical conductivity (EC1) were measured in the samples. The samples were then heated in a boiling water bath for 20 min. After the temperature of the samples reached 25°C, the electrical conductivity (EC2) was again measured. The MTI is expressed as follows: MTI = (1 − (EC1/EC2)) × 100.

### 2.8 Determination of plant Pb content

The Pb content in plant shoots and roots was determined according to the procedure described by Wang et al. (2009). After washing with deionized water, the shoot and root samples were dried at 75°C for 24 hr. Then, 0.1 g of ground shoots and roots of dried samples were digested in 10 ml HNO_3_:HClO_4_ solution and heated in an oven at 100–200°C until near dryness. Subsequently, 5 ml of 5% HNO_3_ was added to dissolve the cooled residue in addition to ddH_2_O to a volume of 20 ml. ICP‐MS (Agilent ICP‐MS 7700ce, Agilent Technologies) was used to determine the plant Pb content.

### 2.9 Determination of root metabolic activity (RA)

The RA was measured according to the method of Liu, Wei, and Li (2014). Briefly, 0.5 g fresh root sample was immersed in 10 ml of a mixed liquid of 0.4% TTC (2,3,5‐triphenyitetrazolium chloride) and 66 mmol/L phosphate buffer solution. The reaction solution was maintained at 37°C for 3 hr, followed by the addition of 2 ml sulfuric acid (1 mol/L) to terminate the reaction. The root was removed and ground in 2 ml ethyl acetate to extract TTF (1,3,5‐triphenylformazan). The absorbance of the extract was measured at a wavelength of 485 nm.

### 2.10 Plant growth parameters

At the end of the experiment, the plant height (PH), leaf area (LA), leaf numbers per plant (LN), and tiller numbers per plant (TN) of each species subjected to each Pb treatment were measured. The plant height, LN, and TN of each plant were determined prior to harvest. The LA was determined based on the leaf area meter (CI 202, rea meter, CID Incorporated).

### 2.11 Plant harvest

After 6 weeks of cultivation, all plants were harvested. The plant biomass was separated into two parts: the root biomass and aboveground biomass. The plant material (leaves, stems and roots) was heated in an oven at 105°C for 30 min and dried at 75°C for 48 hr. After the weight of the samples reached a constant value, their dry weight was recorded.

### 2.12 Bioconcentration factor (BCF) and translocation factor (TF)

The BCF and TF were calculated to evaluate the accumulation of Pb in plants and the translocation from roots to aboveground tissue, respectively. BCF was calculated by dividing the Pb concentration of the root biomass by the Pb concentration of the soil. TF was calculated by dividing the Pb concentration of the plant shoot by the Pb concentration of plant root.

### 2.13 Calculation of comprehensive evaluation value (CEV)

The CEV was used to figure out the most sensitive/tolerant ornamental plant species under different soil Pb treatments. The development of CEV was based on the combination of the cluster analysis and standard deviation coefficient allocation weighted method described by Zhong et al. (2019) with modifications. Four steps were followed to calculate the CEV.

1. Principal component analysis

Principal component analysis (PCA) was applied to extract a common factor with a cumulative variance contribution rate ≥ 85%. The factor scores (FCs) of standardized parameters were calculated. The variance contribution rate of the principal component was set as the index weight (*W*) for each common factor. The principal component score (PCS) could then be calculated by FC multiplied by *W*.

1. Calculation of the subordinate function value

The PCS was normalized according to a subordinator function (see Equations 1 and 2) to obtain the subordinate function value (SFV).

(1) $$\text{SFV}\left({\text{PCS}}_{\mathrm{ij}}\right)=\frac{{\text{PCS}}_{\mathrm{ij}}-{\text{PCS}}_{\min}}{{\text{PCS}}_{\max}-{\text{PCS}}_{\min}}$$

(2) $$\text{SFV}\left({\text{PCS}}_{\mathrm{ij}}\right)=\frac{{\text{PCS}}_{\max}-{\text{PCS}}_{\mathrm{ij}}}{{\text{PCS}}_{\max}-{\text{PCS}}_{\min}}$$

where SFV(PCS*_ij_*) is the subordinate function value among the PCS*_ij_* values for the jth variable, PCS*_ij_* is the value of the principal component score among the PCS*_ij_* values for the *j*th variable, *i* represents different plant genotypes, *j* represents different Pb treatments, PCS_max_ denotes the maximal value among the PCS*_ij_* values for the *j*th variable, and PCS_min_ denotes the minimal value among the PCS*_ij_* values for the *j*th variable. If the measured parameter had a positive relationship with plant Pb tolerance, the subordinator function should be expressed by Equation (1). In contrast, if there is a negative relationship between the measured parameter and plant Pb tolerance, Equation (2) should be used.

1. Calculation of the comprehensive evaluation value

The comprehensive evaluation value was calculated using Equation (3):

(3) $${\text{CEV}}_{i}=\sum \text{SFV}\left({\text{PCS}}_{\mathrm{ij}}\right)\times W_{i}$$

where *W_i_* is the factor weight, which is the ratio of the index weight of each extracted score to the weighted summations of all extracted scores in the *i*th plant.

1. Reorder of the comprehensive evaluation value

With the calculated comprehensive evaluation value, the responses of different ornamental genotypes to Pb stress could be reflected by a single value. The values of five plant species were arranged in descending order and labelled a, b, c, d, and e, respectively, where a denotes the most Pb‐tolerant and e denotes the most Pb‐sensitive plant species.

### 2.14 Statistical analysis

**FIGURE 1:**
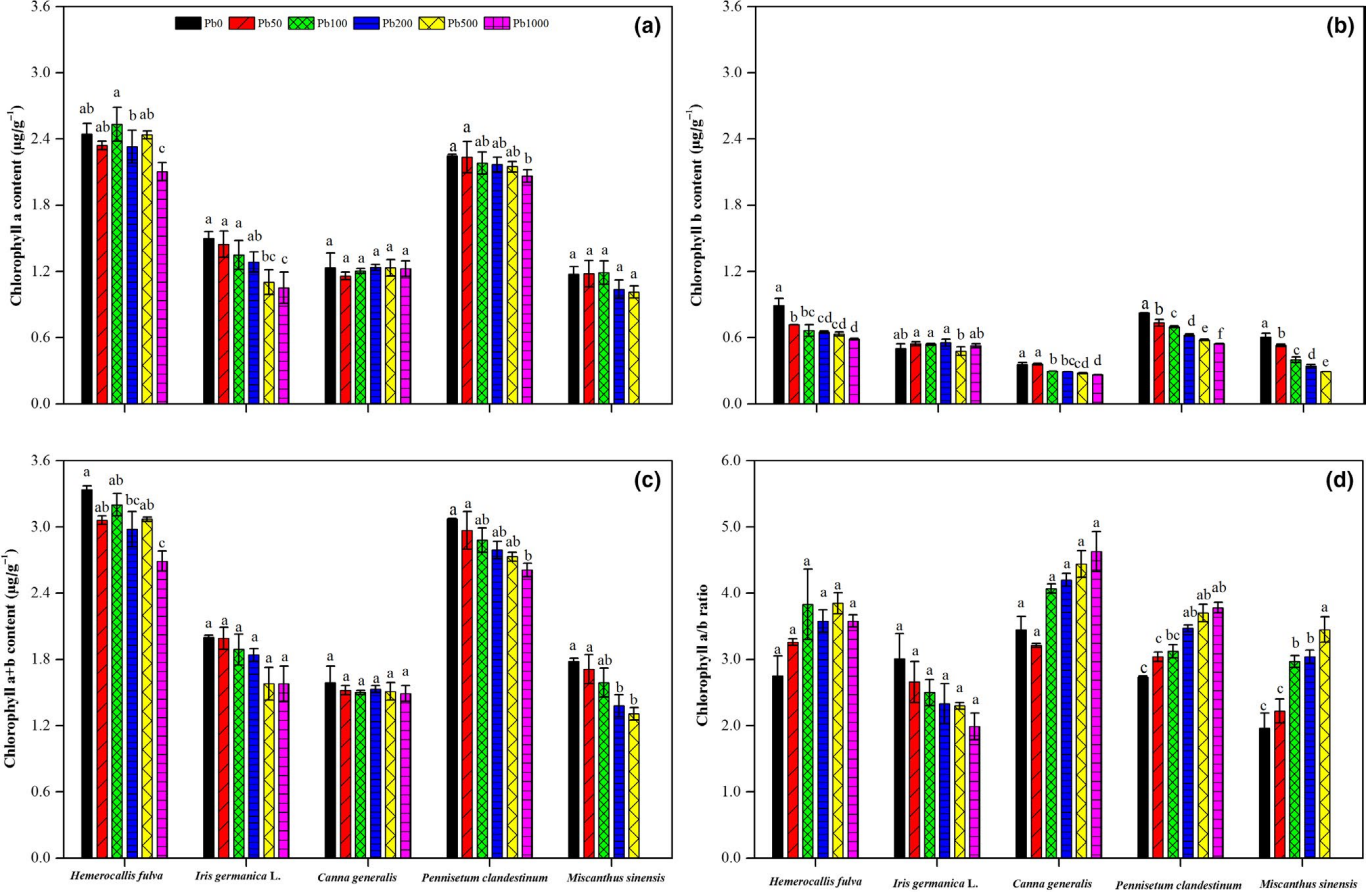
Effects of Pb treatment on chlorophyll *a* (a), chlorophyll *b* (b), chlorophyll *a* + *b* (c), and chlorophyll *a*/*b* (d) in leaves of *Hemerocallis fulva*, *Iris germanica* L., *Canna generalis*, *Pennisetum clandestinum,* and *Miscanthus sinensis*. Vertical bars represent ± *SD* of the mean (*n* = 3); different letters on the *SD* bars indicate significant differences among the Pd treatments (*p* < .05). Pb, Pb treatment; PS, plant species. **Significant differences at *p* < .01

**FIGURE 2:**
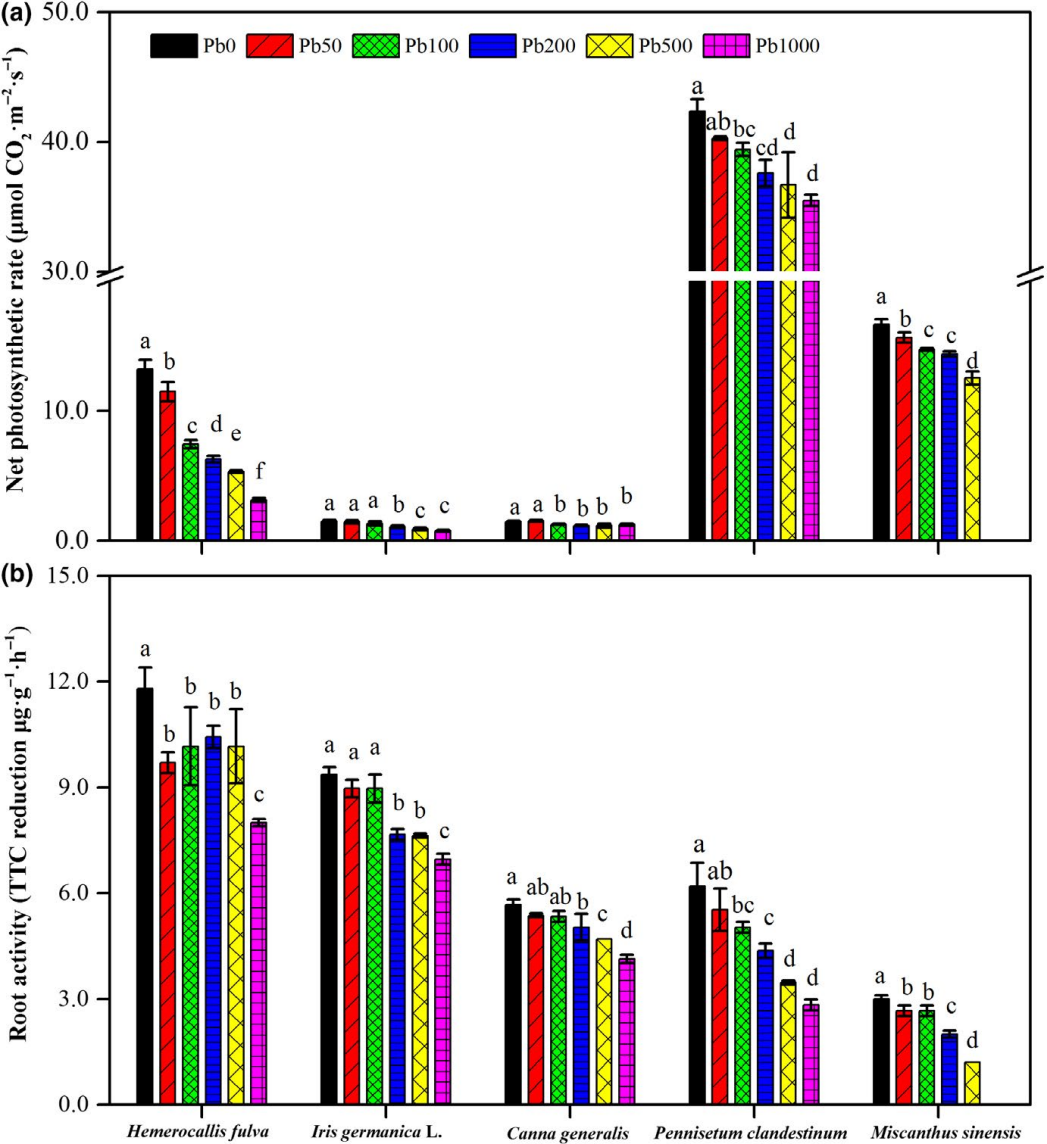
Effects of Pb treatment on leaf net photosynthetic rate (a) and root activity (b) of *Hemerocallis fulva*, *Iris germanica* L., *Canna generalis*, *Pennisetum clandestinum*, and *Miscanthus sinensis*. Vertical bars represent ± *SD* of the mean (*n* = 3); different letters on the *SD* bars indicate significant differences among the Pd treatments (*p* < .05). Pb, Pb treatment; PS, plant species. **Significant differences at *p* < .01

**FIGURE 3:**
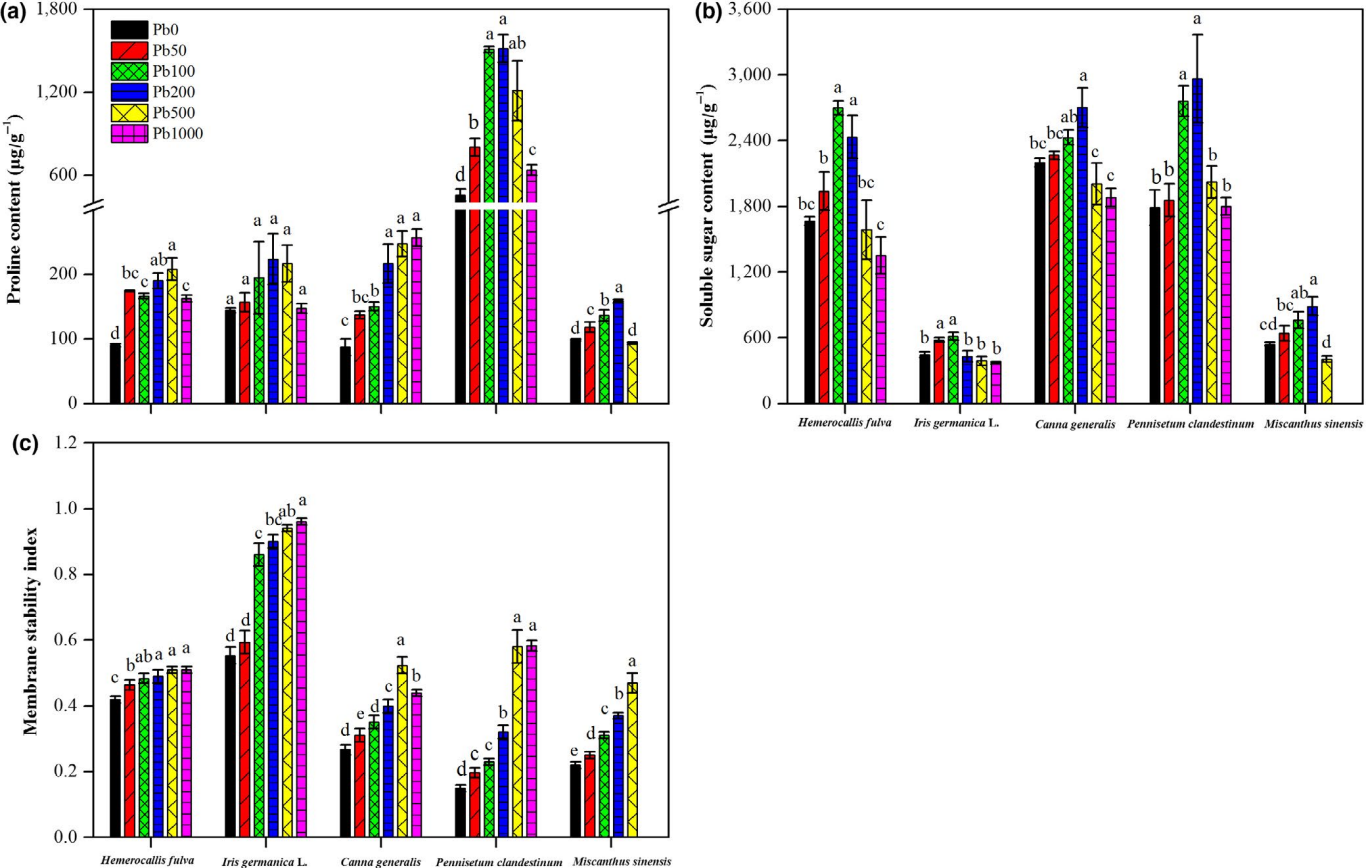
Effects of Pb treatment on proline (a), soluble sugar (b) and membrane stability index (c) in leaves of *Hemerocallis fulva*, *Iris germanica* L., *Canna generalis*, *Pennisetum clandestinum,* and *Miscanthus sinensis*. Vertical bars represent ± *SD* of the mean (*n* = 3); different letters on the *SD* bars indicate significant differences among the Pd treatments (*p* < .05). Pb, Pb treatment; PS, plant species. **Significant differences at *p* < .01

**FIGURE 4:**
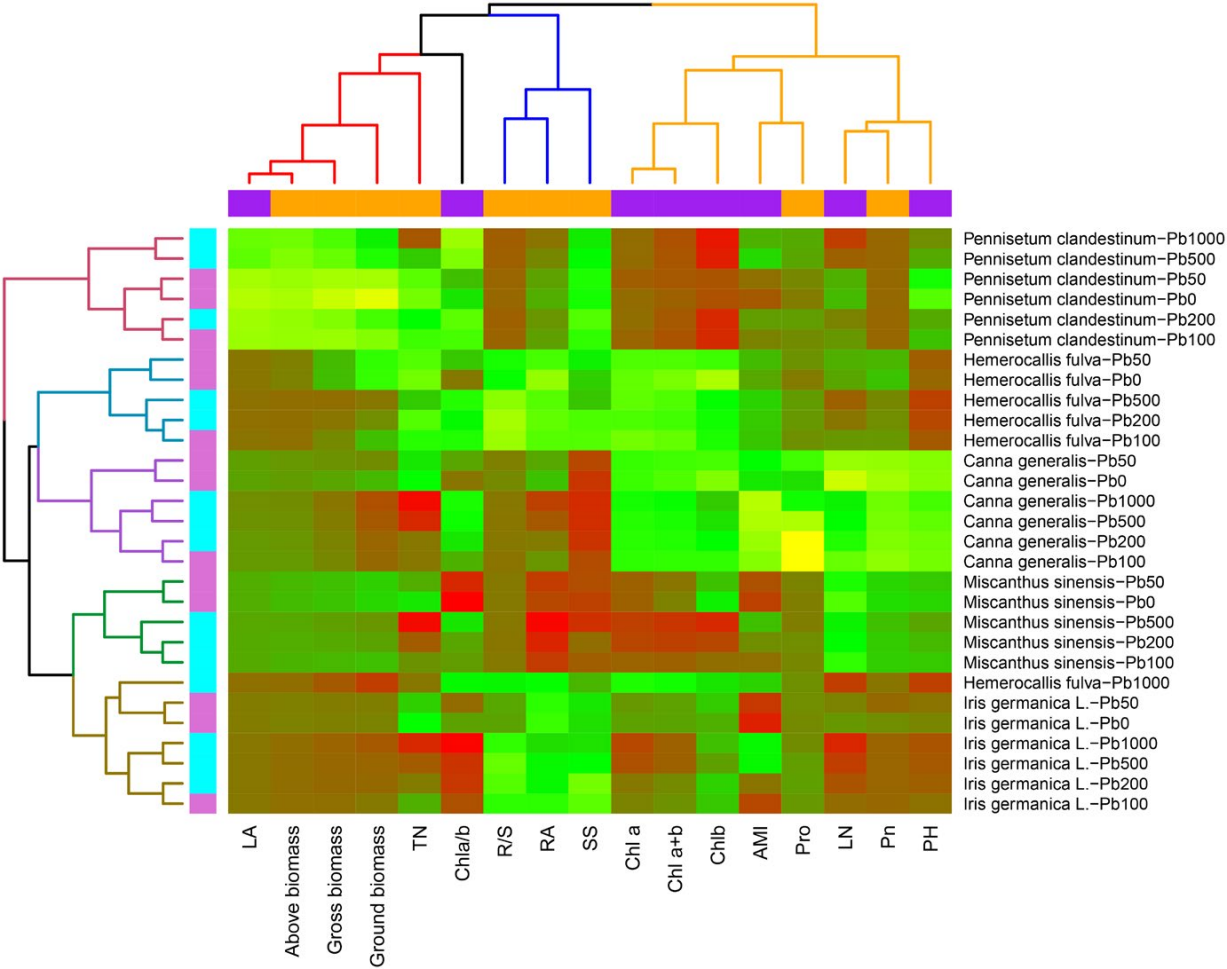
Heatmap based on the Spearman correlation matrix of the 17 variables (plant physiological and biochemical parameters) in different Pb‐treated ornamental plants. AB, above biomass of plant; Chl *a* + *b*, chlorophyll *a* + chlorophyll *b*; Chl *a*/*b*, chlorophyll *a*/chlorophyll *b*; Chla, chlorophyll *a*; Chlb, chlorophyll *b*; GB, gross biomass of plant; LA, leaf area; LN, leaf number per plant; MTI, membrane thermostability index; PH, plant height; *P*_n_, net photosynthetic rate; Pro, proline; R/S, root biomass/aboveground biomass; RA, root activity; RB, root biomass of plant; SS, soluble sugar; TN, tiller number per plant

The experiment was conducted using a completely randomized block design with three replicates. All the parameters described above were measured at six weeks after the plants were subjected to soil Pb treatments. The data for *Miscanthus sinensis* exposed to Pb1000 treatment are not shown in the study because this plant could not survive under these severe Pb stress conditions. The SPSS 18.0 statistical software package (SPSS) was used to perform the statistical analyses. The mean with standard deviation (±*SD*) is shown for each treatment in the tables and figures. The parameters were analyzed by one‐/two‐way analysis of variance (ANOVA) followed by Duncan's multiple range test at *p* < .05. Figures 1, 2 and 3 were performed with the help of Origin 9.0 software (Origin Lab). The heatmap in Figure 4 was constructed by R 3.6.1 (Bell Laboratories).

## 3 RESULTS

### 3.1 Plant growth and biomass

The changes in plant growth parameters (c.v. PH, LN, LA, and TN) and biomass (c.v. AB, RB, GB, and R/S) in different ornamental plants under six soil Pb treatments are shown in Table 1. For *Hemerocallis fulva*, Pb stress significantly decreased PH, LN, LA, TN, AB, RB, and GB. As for the R/S ratio of *Hemerocallis fulva*, the Pb50 and Pb1000 treatment had no significant difference with the Pb0 treatment, but the other Pb treatments (Pb100, Pb200 and Pb500) significantly increased the R/S ratio by 74.6%–84.6%. For *Iris germanica* L., different Pb treatments significantly decreased PH by 2.2%–38.1%, LN by 3.7%–20.2%, TN by 11.3%–49.1%, AB by 3.5%–20.1%, RB by 4.7%–28.8%, and GB by 3.9%–24.5. However, Pb stress showed no significant effect on the LA and the R/S ratio in *Iris germanica* L. All the plant growth parameters (except for LA) and biomass of *Canna generalis* were significantly affected by Pb stress and decreased with increasing Pb in the soil. Excluding the Pb treatments that had no significant effect on LN in *Pennisetum clandestinum* and PH and LN in *Miscanthus sinensis*, the changes in other parameters in *Pennisetum clandestinum* and *Miscanthus sinensis* under different Pb treatments exhibited similar trends to *Canna generalis*.

**TABLE 1:** Effects of different Pb treatments on the plant growth and biomass of *Hemerocallis fulva*, *Iris germanica* L., *Canna generalis*, *Pennisetum clandestinum*, and *Miscanthus sinensis*

Plant species	Treatment	PH (cm)	LN	LA (cm^−2^· plant)	TN	AB (g)	RB (g)	GB (g)	R/S
*Hemerocallis fulva*	P0	16.95 ± 1.24 a	16.7 ± 0.33 a	39.5 ± 1.83 a	6.7 ± 0.21 a	6.7 ± 0.5 a	41.2 ± 2.0 a	47.9 ± 2.7 a	6.15 ± 0.46 b
	P50	14.78 ± 2.25 a	17.0 ± 0.58 a	38.5 ± 4.44 ab	6.3 ± 0.88 a	6.1 ± 0.4 a	40.7 ± 0.1 a	46.8 ± 2.2 a	6.63 ± 0.17 b
	P100	13.76 ± 1.34 a	16.3 ± 0.33 ab	34.8 ± 3.64 ab	5.7 ± 0.33 ab	2.4 ± 0.3 b	26.6 ± 2.3 b	29.0 ± 3.2 b	11.07 ± 0.33 a
	P200	9.91 ± 1.26 b	15.3 ± 0.33 bc	29.1 ± 1.38 ab	6.3 ± 0.3 a	1.8 ± 0.5 b	20.3 ± 2.3 c	22.1 ± 2.3 c	11.54 ± 0.57 a
	P500	8.28 ± 2.28 b	14.7 ± 0.33 cd	29.6 ± 2.7 ab	4.7 ± 0.88 bc	1.6 ± 0.2 b	17.3 ± 2.1 c	18.9 ± 2.4 c	10.74 ± 1.06 a
	P1000	7.97 ± 2.21 b	13.7 ± 0.67 d	28.6 ± 1.88 b	3.7 ± 0.33 c	1.8 ± 0.4 b	10.1 ± 0.9 d	11.9 ± 2.2 d	5.53 ± 0.56 b
*Iris germanica* L.	P0	24.08 ± 1.24 a	16.3 ± 0.89 a	53.3 ± 0.65 a	5.3 ± 0.33 a	16.9 ± 0.7 a	19.1 ± 1.2 a	35.9 ± 1.6 a	1.12 ± 0.07 a
	P50	20.02 ± 2.18 b	15.7 ± 0.67 ab	51.4 ± 1.72 a	4.7 ± 0.33 ab	16.3 ± 0.5 ab	18.2 ± 0.6 ab	34.5 ± 1.4 ab	1.12 ± 0.06 a
	P100	19.44 ± 3.22 b	15.0 ± 0.58 abc	45.5 ± 3.44 a	4.3 ± 0.88 abc	15.8 ± 0.2 ab	17.6 ± 0.5 b	33.4 ± 1.1 b	1.11 ± 0.05 a
	P200	15.44 ± 2.75 c	14.3 ± 0.3 bcd	44.9 ± 4.06 a	3.7 ± 0.33 abc	15.1 ± 0.6 abc	15.7 ± 0.4 c	30.8 ± 0.8 c	1.04 ± 0.07 a
	P500	14.10 ± 1.10 c	13.7 ± 0.33 cd	44.2 ± 2.83 a	3.3 ± 0.33 bc	14.5 ± 0.48 bc	15.0 ± 0.4 c	29.5 ± 1.7 c	1.04 ± 0.08 a
	P1000	12.67 ± 1.37 c	13.0 ± 0.58 d	43.6 ± 5.08 a	2.7 ± 0.67 c	13.5 ± 0.5 c	13.6 ± 0.3 d	27.1 ± 0.4 d	1.01 ± 0.07 a
*Canna generalis*	P0	83.52 ± 3.54 a	17.3 ± 0.67 a	683.3 ± 20.77 a	6.7 ± 0.67 a	80.2 ± 2.9 a	63.7 ± 1.4 a	143.9 ± 2.1 a	0.79 ± 0.02 a
	P50	82.17 ± 2.80 ab	17.0 ± 0.58 a	657.3 ± 46.32 a	6.3 ± 0.33 ab	76.3 ± 0.5 ab	55.4 ± 1.2 b	131.7 ± 3.7 b	0.73 ± 0.00 b
	P100	79.14 ± 6.11 ab	16.3 ± 0.33 ab	631.3 ± 27.69 a	6.0 ± 0.58 ab	75.3 ± 0.2 ab	51.4 ± 2.2 b	126.7 ± 0.7 c	0.68 ± 0.00 c
	P200	77.66 ± 7.53 ab	15.7 ± 0.33 bc	640.6 ± 38.65 a	5.3 ± 0.67 ab	74.9 ± 0.5 ab	43.3 ± 2.8 c	118.2 ± 3.4 d	0.58 ± 0.04 d
	P500	73.83 ± 3.80 bc	14.7 ± 0.33 cd	508.6 ± 38.97 b	4.7 ± 0.33 bc	70.3 ± 2.8 bc	38.4 ± 0.9 cd	108.7 ± 1.5 e	0.55 ± 0.00 d
	P1000	67.75 ± 4.40 c	13.7 ± 0.33 d	531.4 ± 6.33 b	3.3 ± 0.33 c	65.7 ± 3.4 c	32.8 ± 4.8 d	98.5 ± 3.3 f	0.50 ± 0.02 e
*Pennisetum clandestinum*	P0	72.222.22 a	25.3 ± 0.88 a	134.78 ± 12.66 a	5.3 ± 0.88 a	13.3 ± 1.3 a	25.7 ± 2.2 a	39.0 ± 3.3 a	1.93 ± 0.18 a
	P50	56.86 ± 3.63 b	23.7 ± 1.20 a	123.4 ± 5.06 ab	5.0 ± 1.53 ab	12.3 ± 0.3 ab	20.0 ± 1.2 b	32.3 ± 1.6 b	1.62 ± 0.05 b
	P100	32.42 ± 2.42 d	22.3 ± 0.67 a	114.1 ± 6.09 ab	3.7 ± 0.67 abc	12.7 ± 0.9 ab	15.5 ± 1.3 c	28.2 ± 2.3 c	1.22 ± 0.05 c
	P200	37.83 ± 2.84 c	20.3 ± 2.73 a	111.6 ± 6.09 ab	3.7 ± 0.88 abc	11.5 ± 0.54 abc	15.2 ± 0.8 c	26.7 ± 1.7 cd	1.32 ± 0.05 c
	P500	32.20 ± 2.20 d	19.3 ± 2.67 a	94.8 ± 7.02 bc	2.7 ± 0.67 bc	10.4 ± 0.5 bc	13.6 ± 0.8 c	24.0 ± 1.8 de	1.31 ± 0.09 c
	P1000	25.93 ± 1.95 e	19.0 ± 3.00 a	87.2 ± 1.29 c	2.3 ± 0.33 c	9.3 ± 0.3 c	12.4 ± 0.6 c	21.7 ± 1.3 e	1.34 ± 0.04 c
*Miscanthus sinensis*	P0	35.59 ± 15.45 a	21.7 ± 2.85 a	152.9 ± 6.48 a	5.0 ± 0.58 a	21.2 ± 0.9 a	29.3 ± 2.0 a	50.4 ± 5.1 a	1.40 ± 0.11 a
	P50	42.67 ± 3.23 a	21.0 ± 2.52 a	152.1 ± 3.21 ab	4.3 ± 0.88 ab	20.0 ± 0.5 a	28.6 ± 0.7 a	48.6 ± 1.8 a	1.43 ± 0.03 a
	P100	39.68 ± 3.21 a	20.7 ± 2.85 a	146.6 ± 6.09 ab	4.0 ± 0.58 ab	19.1 ± 0.3 ab	26.6 ± 1.2 ab	45.7 ± 1.3 a	1.39 ± 0.01 ab
	P200	36.16 ± 4.16 a	19.3 ± 3.18 a	143.2 ± 7.00 b	3.3 ± 0.33 ab	17.9 ± 0.93 b	23.0 ± 0.7 bc	40.9 ± 0.9 b	1.28 ± 0.03 b
	P500	31.41 ± 3.22 a	19.0 ± 2.52 a	140.3 ± 10.53 b	2.3 ± 0.88 b	16.0 ± 0.8 c	21.2 ± 0.4 c	37.2 ± 1.1 b	1.32 ± 0.03 ab

Note

Values are the mean of three replicates. Different letters indicate that values are significantly different from each other at *p* ≤ .05.

### 3.2 Chlorophyll content

The chlorophyll contents of *Hemerocallis fulva*, *Iris germanica* L., *Canna generalis*, *Pennisetum clandestinum*, and *Miscanthus sinensis* are shown in Figure 1. Different Pb treatments had no noticeable effect on Chl *a* of *Canna generalis* and *Miscanthus sinensis* (Figure 1a). For *Hemerocallis fulva* and *Pennisetum clandestinum*, compared with the control, Chl *a* was not significantly impacted by the Pb50, Pb100, Pb200, and P500 treatments, but it was decreased by 13.9% and 8.1% in response to Pb1000, respectively. For *Iris germanica* L., Chl *a* was not significantly affected by the Pb50, Pb100 and Pb200 treatments, but it decreased significantly by 26.4% upon Pb500 treatment and by 29.8% upon Pb1000 treatment. The content of Chl *b* was much lower than Chl *a* in all the plants (Figure 1b). Excluding Chl *b* in leaves of *Iris germanica* L., soil Pb treatments significantly reduced Chl *b* in the leaves of the other four plants. Compared with Pb0, Pb stress decreased the levels of Chl *b* by 19.6%–34.2% in *Hemerocallis fulva*, 17.1%–25.8% in *Canna generalis*, 10.7%–33.7% in *Pennisetum clandestinum* and 12.0%–51.3% in *Miscanthus sinensis*, respectively. Although Chl *a* + *b* decreased in all plants with the increase in Pb content in soil, no significant differences were observed in Pb‐treated *Iris germanica* L. and *Canna generalis* (Figure 1c). For *Hemerocallis fulva*, *Pennisetum clandestinum,* and *Miscanthus sinensis*, compared to untreated plants, Pb50 through Pb1000 treatments decreased Chl *a* + *b* by 4.1%–19.4%, 3.3%–15.0%, and 3.9%–26.6%, respectively. Regarding Chl *a*/*b*, compared with Pb0, increasing Pb stress enhanced the values in *Hemerocallis fulva*, *Canna generalis*, *Pennisetum clandestinum*, and *Miscanthus sinensis* but decreased the values in *Iris germanica* L. (Figure 1d).

### 3.3 Leaf net photosynthetic rate and root activity

The leaf net photosynthetic rate and root activity of *Hemerocallis fulva*, *Iris germanica* L., *Canna generalis*, *Pennisetum clandestinum,* and *Miscanthus sinensis* in response to various soil Pb treatments are illustrated in Figure 2. The values obtained for the *net* photosynthetic rate in leaves of different plants revealed noticeable differences (Figure 2a). Under the Pb0 condition, the highest *P*_n_ value of 13.2 μmol CO_2_ m^−2^ s^−1^ was found in leaves of *Pennisetum clandestinum* and the lowest value of 1.5 μmol CO_2_ m^−2^ s^−1^ in leaves of *Iris germanica* L. and *Canna generalis*, respectively. Under Pb stress conditions, the *P*_n_ values in the leaves of five plants decreased with increasing levels of Pb in the soil. Compared with Pb0, the Pb treatments decreased *P*_n_ in the leaves of *Hemerocallis fulva*, *Iris germanica* L., *Canna generalis*, *Pennisetum clandestinum*, and *Miscanthus sinensis* by 13.1%–76.2%, 3.4%–78.2%, 13.4%–20.0%, 4.9%–16.2%, and 6.0%–24.6%, respectively.

The root activities in *Hemerocallis fulva*, *Iris germanica* L., *Canna generalis*, *Pennisetum clandestinum,* and *Miscanthus sinensis* were significant effected by Pb stress and decreased with the increasing of Pb contents in the soil (Figure 2b). The lowest RA values of *Hemerocallis fulva*, *Iris germanica* L. *Canna generalis*, and *Pennisetum clandestinum* in the Pb1000 treatment were 8.0 μg g^−1^ hr^−1^, 7.0 μg g^−1^ hr^−1^, 4.1 μg g^−1^ hr^−1^, and 2.8 in μg g^−1^ hr^−1^. The lowest RA values of *Miscanthus sinensis* in the Pb500 treatment was 1.2 μg g^−1^ hr^−1^.

### 3.4 Proline, soluble sugar and membrane stability index

Changes in Pro, SS and MTI in leaves of *Hemerocallis fulva*, *Iris germanica* L., *Canna generalis*, *Pennisetum clandestinum,* and *Miscanthus sinensis* are shown in Figure 3. For *Iris germanica* L., although Pb stress increased Pro by 1.9%–50.4%, the difference in Pro following the different Pb treatments was not significant. For the other plants, compared with Pb0, Pb stress significantly increased Pro by 80.5%–130.8 in *Hemerocallis fulva*, 56.8%–193.9% in *Canna generalis*, and 3.9%–231.6% in *Pennisetum clandestinum*. The change in Pro in *Miscanthus sinensis* was small. Pb50, Pb100, and Pb200 treatment significantly increased Pro in *Miscanthus sinensis*, where Pb500 treatment decreased Pro by 4.9% compared with Pb0 treatment.

The content of SS in all plants showed similar changes (Figure 3b). SS significantly increased in the presence of a low soil Pb content (50–200 mg/kg) but not a high soil Pb content (500–1,000 mg/kg). Compared with the Pb0 treatment, the Pb50, Pb100, and Pb200 treatments increased SS by 16.4%, 62.1% and 46.1% in *Hemerocallis fulva*, 3.1%, 37.2%, and 22.9% in *Canna generalis* and 18.1%, 40.4, and 63.6% in *Miscanthus sinensis*. On the other hand, compared with the Pb0 treatment, the Pb500 and Pb1000 treatments decreased SS by 4.7% and 18.8% in *Hemerocallis fulva*, and by 8.8% and 14.4% in *Canna generalis*. As for *Miscanthus sinensis*, SS decreased by 25.6% in the Pb500 treatment. For *Pennisetum clandestinum*, all soil Pb treatments enhanced SS by 0.6%–65.9%. For *Iris germanica* L., SS increased to 134.0 μg/g following Pb50 treatment and 166.7 μg/g following Pb100 treatment, but it decreased to 16.6 μg/g in response to Pb200 treatment, 57.5 μg/g in response to Pb500 treatment and 76.1 μg/g in response to Pb1000 treatment, respectively.

MTI was significantly influenced in all plants by soil Pb stress and increased with increasing levels of Pb in the soil (Figure 3c). The highest values of MTI were 0.51 in *Hemerocallis fulva*, 0.96 in *Iris germanica* L., and 0.58 in *Pennisetum clandestinum* in response to Pb1000 treatment and 0.52 in *Canna generalis* and 0.47 in *Miscanthus sinensis* in response to Pb500 treatment, respectively.

### 3.5 Concentration of Pb in plant tissues

Soil Pb stress notably enhanced the Pb concentration either in shoots or roots of *Hemerocallis fulva*, *Iris germanica* L., *Canna generalis*, *Pennisetum clandestinum,* and *Miscanthus sinensis* (Table 2). In non‐Pb‐contaminated soils, the lowest Pb concentrations were detected in shoots (9.64 μg/g DW) and roots (13.9 μg/g DW) of *Miscanthus sinensis*. In contrast, *Canna generalis* had the highest shoot and root Pb concentrations, which were four‐fold those of *Miscanthus sinensis*. Under Pb stress conditions, the Pb contents in plant shoots and roots increased with Pb stress, and the highest values were detected in response to Pb1000 treatment in *Hemerocallis fulva*, *Iris germanica* L., *Canna generalis*, and *Pennisetum clandestinum,* and in response to Pb500 treatment in *Miscanthus sinensis*. Moreover in response to the same Pb treatment, the concentrations of Pb in shoots (110.74 μg/g DW ~ 1,032.76μg/g) and roots (151.34–1,904.33 μg/g) were much higher in *Canna generalis* than other plants.

**TABLE 2:** Concentrations of Pb in shoots and roots of *Hemerocallis fulva*, *Iris germanica* L., *Canna generalis*, *Pennisetum clandestinum* and *Miscanthus sinensis*

Plant species	Pb treatments	Pb concentrations in shoot (μg/g DW)	Pb concentrations in root (μg/g DW)	BCF	TF
*Hemerocallis fulva*	Pb0	12.01 ± 0.12 f	20.13 ± 0.43 f		0.60 ± 0.01 a
	Pb50	35.24 ± 1.77 e	66.49 ± 0.44 e	0.70 ± 0.0a a	0.53 ± 0.03 b
	Pb100	65.65 ± 0.76 d	129.10 ± 2.26 d	0.66 ± 0.01 a	0.51 ± 0.01 b
	Pb200	102.61 ± 2.17 c	229.75 ± 3.00 c	0.51 ± 0.01 b	0.45 ± 0.01 c
	Pb500	203.37 ± 2.02 b	507.07 ± 13.29 b	0.41 ± 0.01 c	0.40±0.01 d
	Pb1000	301.42 ± 6.37 a	788.32 ± 7.84 a	0.30 ± 0.02 d	0.38 ± 0.01 d
*Iris germanica* L.	Pb0	17.13 ± 0.62 f	22.47 ± 0.24 f		0.76 ± 0.03 a
	Pb50	25.17 ± 0.89 e	42.81 ± 0.40 e	0.50 ± 0.02 a	0.59 ± 0.02 b
	Pb100	42.11 ± 0.68 d	75.31 ± 0.96 d	0.42 ± 0.01 b	0.56 ± 0.01 b
	Pb200	72.78 ± 0.60 c	142.05 ± 2.05 c	0.36 ± 0.01 bc	0.51 ± 0.02 c
	Pb500	162.85 ± 1.97 b	336.29 ± 5.23 b	0.33 ± 0.01 cd	0.48 ± 0.01 cd
	Pb1000	251.67 ± 3.43 a	561.28 ± 2.24 a	0.25 ± 0.01 d	0.45 ± 0.01 d
*Canna generalis*	Pb0	40.66 ± 1.18 f	51.15 ± 1.52 f		0.80 ± 0.01 a
	Pb50	110.74 ± 2.24 e	151.34 ± 3.09 e	2.21 ± 0.04 a	0.73 ± 0.02 b
	Pb100	199.83 ± 1.54 d	287.34 ± 3.00 d	2.00 ± 0.02 b	0.69 ± 0.01 c
	Pb200	312.93 ± 6.98 c	485.18 ± 8.17 c	1.56 ± 0.04 c	0.64 ± 0.01 d
	Pb500	694.79 ± 10.67 b	1,215.25 ± 27.86 b	1.39 ± 0.02 c	0.57 ± 0.01 e
	Pb1000	1,032.76 ± 15.98 a	1,904.33 ± 25.66 a	1.03 ± 0.02 d	0.54 ± 0.01 f
*Pennisetum clandestinum*	Pb0	13.41 ± 0.03 f	22.27 ± 0.28 f		0.60 ± 0.01 a
	Pb50	20.63 ± 0.33 e	40.24 ± 0.49 e	0.41 ± 0.01 a	0.51 ± 0.01 b
	Pb100	28.83 ± 1.06 d	60.55 ± 2.42 d	0.29 ± 0.01 b	0.48 ± 0.01 c
	Pb200	39.72 ± 1.45 c	96.49 ± 1.47 c	0.20 ± 0.01 c	0.41 ± 0.02 d
	Pb500	76.72 ± 1.44 b	199.62 ± 3.86 b	0.15 ± 0.01 d	0.39 ± 0.01 d
	Pb1000	85.15 ± 0.73 a	249.45 ± 7.75 a	0.09 ± 0.01 e	0.34 ± 0.01 e
*Miscanthus sinensis*	Pb0	9.64 ± 0.27 d	13.90 ± 0.33 d		0.69 ± 0.01 a
	Pb50	11.23 ± 0.19 d	17.27 ± 0.31 d	0.55 ± 0.01 a	0.65 ± 0.02 a
	Pb100	14.64 ± 0.83 c	30.44 ± 1.22 c	0.42 ± 0.01 b	0.48 ± 0.01 b
	Pb200	21.97 ± 0.30 b	50.72 ± 0.66 b	0.23 ± 0.01 c	0.43 ± 0.01 b
	Pb500	49.49 ± 0.87 a	131.13 ± 2.99 a	0.12 ± 0.01 d	0.38 ± 0.01 c

Note

Values are the mean of three replicates. Different letters indicate that values are significantly different from each other at *p* ≤ .05.

The calculated BCF and TF values in *Hemerocallis fulva*, *Iris germanica* L., *Canna generalis*, *Pennisetum clandestinum,* and *Miscanthus sinensis* decreased with the increasing Pb contents in the soil (Table 3). Excluding the BCF in *Canna generalis*, which ranged from 2.21 to 1.03, all the BCFs in the other four plants were <1.0. The TFs in all five plants were <1.0, and the TF values decreased from 0.60 to 0.38 in *Hemerocallis fulva*, 0.76 to 0.45 in *Iris germanica* L., 0.80 to 0.54 in *Canna generalis*, 0.60 to 0.34 in *Pennisetum clandestinum*, and 0.69 to 0.38 in *Miscanthus sinensis*, respectively.

**TABLE 3:** Multiple range test of the effects of soil Pb stress and plant species on plant physiological and biochemical parameters based on one‐/two‐way ANOVA

Plant parameters	Pb stress			Plant species			Pb stress × Plant species		
	*df*	*F*	*p*	*df*	*F*	*p*	*df*	*F*	*p*
PH	4	664.9	.00	5	23.5	.00	19	11.3	0
LN	4	64.1	.00	5	13.0	.00	19	0.6	.924
LA	4	4,215.6	.00	5	26.0	.00	19	10.9	.00
TN	4	103.7	.00	5	114.2	.00	19	2.0	.021
Above biomass	4	15,695.4	.00	5	87.0	.00	19	10.3	.000
Root biomass	4	1,117.0	.00	5	278.0	.00	19	29.2	.000
Gross biomass	4	5,582.3	.00	5	246.2	.00	19	22.4	.000
R/S	4	1,790.4	.00	5	27.3	.00	19	19.8	.001
Chl *a*	4	699.2	.00	5	11.6	.00	19	2.9	.000
Chl *b*	4	834.7	.00	5	133.9	.00	19	18.7	.000
Chl *a* + *b*	4	1,045.9	.00	5	34.8	.00	19	3.5	.000
Chl *a*/*b*	4	152.1	.00	5	32.4	.00	19	11.8	.000
RA	4	1,102.1	.00	5	77.7	.00	19	4.4	.000
*P*_n_	4	11,607.2	.00	5	104.4	.00	19	21.1	.000
Pro	4	921,766.0	.00	5	66,364.8	.00	19	41,186.1	.000
SS	4	1,454.4	.00	5	153.8	.00	19	18.4	.000
MTI	4	1,597.2	.00	5	471.2	.00	19	44.7	.000

### 3.6 Analysis of variance on plant parameters

The effects of different Pb treatments, plant species and their interactions on PH, LN, LA, TN, AB, RB, TB, R/S, Chl *a*, Chl *b*, Chl *a* + *b*, Chl *a*/*b*, RA, Pn, Pro, SS, and MSI are shown in Table 3. All the measured parameters were significantly affected (*p* < .05) by the Pb treatments or the plant species. The interactive effect of the Pb treatments and plants species on LN was not significant (*p* = .924), but it had a notable effect on the other parameters (*p* < .01).

### 3.7 Identification of Pb tolerant plant species

The CEV of the different plants in response to the six Pb treatments are listed in Table 4. Under non‐Pb‐stressed conditions, *Hemerocallis fulva* had the highest value (0.64) and *Iris germanica* L. had the lowest value (0.07). Under Pb50 conditions, the highest value (0.66) and lowest value (0.10) of CEV was detected in *Pennisetum clandestinum* and *Iris germanica* L., respectively. Following treatment with Pb100, Pb200, Pb 500, and Pb1000, *Iris germanica* L. retained the lowest values (0.05, 0.07, 0.08, and 0.12, respectively), but the highest values (0.71, 0.67, 0.72, and 0.67, respectively) were detected in *Canna generalis*.

**TABLE 4:** Comprehensive evaluation of Pb tolerance for five ornamental plant species by principal component analysis (PCA)

Pb treatments	Plant species	FS1	FS2	FS3	W1	W2	W3	PCS1	PCS2	PCS3	SFV1	SFV2	SFV3	CEV	Order
Pb0	*Hemerocallis fulva*	1.52	0.24	−0.89	2.785	2.182	1.844	4.24	0.51	−1.65	1.00	0.53	0.00	0.64	a
	*Iris germanica* L.	−0.57	−1.20	−0.58	2.785	2.182	1.844	−1.58	−2.62	−1.08	0.09	0.00	0.12	0.07	e
	*Canna generalis*	0.52	−0.31	1.68	2.785	2.182	1.844	1.45	−0.69	3.10	0.56	0.32	1.00	0.56	b
	*Pennisetum clandestinum*	−0.70	1.53	−0.01	2.785	2.182	1.844	−1.95	3.34	−0.01	0.03	1.00	0.34	0.39	c
	*Miscanthus sinensis*	−0.78	−0.25	−0.20	2.785	2.182	1.844	−2.16	−0.55	−0.36	0.00	0.35	0.27	0.16	d
Pb50	*Hemerocallis fulva*	−0.40	−0.50	1.41	2.746	2.212	1.977	−1.11	−1.11	2.79	0.11	0.17	1.00	0.35	c
	*Iris germanica* L.	−0.68	−0.96	−0.26	2.746	2.212	1.977	−1.87	−2.12	−0.51	0.00	0.00	0.40	0.10	e
	*Canna generalis*	−0.29	1.67	0.27	2.746	2.212	1.977	−0.79	3.69	0.53	0.16	1.00	0.59	0.52	b
	*Pennisetum clandestinum*	1.77	−0.21	−0.04	2.746	2.212	1.977	4.86	−0.47	−0.09	1.00	0.28	0.48	0.66	a
	*Miscanthus sinensis*	−0.40	0.00	−1.37	2.746	2.212	1.977	−1.09	0.00	−2.73	0.12	0.36	0.00	0.16	d
Pb100	*Hemerocallis fulva*	−0.89	0.14	1.33	7.755	2.379	1.773	−6.90	0.34	2.36	0.00	0.45	1.00	0.35	c
	*Iris germanica* L.	−0.88	−1.04	−0.46	7.755	2.379	1.773	−6.85	−2.48	−0.81	0.00	0.00	0.25	0.05	e
	*Canna generalis*	1.49	−0.24	0.76	7.755	2.379	1.773	11.57	−0.58	1.35	1.00	0.30	0.76	0.71	a
	*Pennisetum clandestinum*	−0.14	1.62	−0.60	7.755	2.379	1.773	−1.10	3.84	−1.06	0.31	1.00	0.18	0.53	b
	*Miscanthus sinensis*	0.42	−0.47	−1.04	7.755	2.379	1.773	3.29	−1.12	−1.84	0.55	0.21	0.00	0.33	d
Pb200	*Hemerocallis fulva*	−0.69	−0.29	1.31	2.656	2.478	1.818	−1.84	−0.71	2.38	0.07	0.24	1.00	0.32	c
	*Iris germanica* L.	−0.87	−0.93	−0.40	2.656	2.478	1.818	−2.32	−2.29	−0.73	0.00	0.00	0.35	0.07	e
	*Canna generalis*	1.59	−0.21	0.58	2.656	2.478	1.818	4.23	−0.52	1.05	1.00	0.27	0.72	0.67	a
	*Pennisetum clandestinum*	−0.34	1.71	−0.16	2.656	2.478	1.818	−0.90	4.24	−0.28	0.22	1.00	0.45	0.55	b
	*Miscanthus sinensis*	0.31	−0.29	−1.33	2.656	2.478	1.818	0.84	−0.73	−2.42	0.48	0.24	0.00	0.30	d
Pb500	*Hemerocallis fulva*	−0.84	−0.31	1.19	2.632	2.393	1.833	−2.20	−0.73	2.19	0.00	0.24	1.00	0.30	d
	*Iris germanica* L.	−0.78	−0.95	−0.45	2.632	2.393	1.833	−2.07	−2.27	−0.83	0.02	0.00	0.34	0.08	e
	*Canna generalis*	1.52	−0.14	0.78	2.632	2.393	1.833	4.00	−0.32	1.43	1.00	0.31	0.84	0.72	a
	*Pennisetum clandestinum*	−0.38	1.70	−0.22	2.632	2.393	1.833	−0.99	4.07	−0.39	0.19	1.00	0.44	0.53	b
	*Miscanthus sinensis*	0.48	−0.31	−1.31	2.632	2.393	1.833	1.26	−0.75	−2.40	0.56	0.24	0.00	0.33	c
Pb1000	*Hemerocallis fulva*	−0.89	−0.42	1.13	2.883	2.630	1.331	−2.57	−1.10	1.50	0.00	0.18	1.00	0.18	c
	*Iris germanica* L.	−0.30	−0.83	−1.21	2.883	2.630	1.331	−0.87	−2.19	−1.61	0.25	0.00	0.00	0.12	d
	*Canna generalis*	1.43	−0.20	0.40	2.883	2.630	1.331	4.13	−0.52	0.53	1.00	0.28	0.69	0.67	a
	*Pennisetum clandestinum*	−0.24	1.45	−0.31	2.883	2.630	1.331	−0.69	3.81	−0.42	0.28	1.00	0.38	0.58	b

Abbreviations: CEV, Comprehensive evaluation value; FS, Factor score obtained by PCA method; PCS, Principal component score; SFV, Subordinate function value; W, Index weight.

## 4 DISCUSSION

Pb toxicity has been studied widely in many higher plants for evaluations of Pb tolerance or a high Pb uptake potential of plants (Andra et al., 2009), aiming to select candidate plants for the phytoremediation of Pb‐contaminated regions (Gupta & Chandra, 1994). Although the negative effect of Pb on plant physiological and biochemical activities has been widely accepted (Jayasri & Suthindhiran, 2017; Sharma & Dubey, 2005; Verma & Dubey, 2003), some other studies have provided opposite conclusions (Chandrasekhar & Ray, 2019). For example, Sidhu, Singh, Batish, and Kohli (2017) reported that soil Pb stress enhanced the growth, protein, and carbohydrate levels of *Coronopus didymus* L. Hou et al. (2018) also found that the maximum photochemical efficiency of photosystem II in *Pogonatherum crinitum* was enhanced by Pb^2+^. The different results indicate that different plant species exhibit a range of responses to the Pb concentration, which strongly depends on the soil characteristics, Pb concentration, stress exposure time, and plant genotype (Gao et al., 2010; Pourrut, Shahid, Dumat, Winterton, & Pinelli, 2011; Shu, Yin, Zhang, & Wang, 2012). Furthermore, the types of Pb compound (e.g., Pb(NO_3_)_2_, PbCl_2_, and (CH_3_COO)_2_Pb·3H_2_O) used in the soil Pb treatments may also lead to a different response to Pb stress (Kurtyka, Burdach, Siemieniuk, & Karcz, 2018; Qin et al., 2018; Verma & Dubey, 2003). According to the the results of One‐way ANOVA (Table 3), our present results revealed that ornamental plants subjected to soil Pb stress showed a pronounced decrease in the growth traits (plant height, leaf area, leaf number and tiller number) and in plant biomass compared with non‐Pb‐treated plants, and reduction in growth, physiology and yield with increasing Pb content in the soil (Table 1) indicated that Pb stress posed a serious threat to the normal growth and metabolic activities of the plants. Similar findings have been reported by Hussain et al. (2013).

The non‐uniform distribution of Pb in different parts of the plants and relatively higher levels of heavy metals in edible tissues have become a great challenge (Han, Gao, Geng, Li, & Wang, 2018; Natasha et al., 2018). In the present study, Pb treatment enhanced the Pb concentration in both plant roots and shoots in all the plants, and the content of Pb increased with increasing levels of Pb in the soil (Table 2). However, although Pb bioaccumulated in all Pb‐stressed plants, the concentration of Pb ion in various plant species and the distribution of Pb in different tissues in the same plant were different. As shown in Table 2, the Pb concentration was much higher in *Canna generalis* than in the other four plant species, indicating that *Canna generalis* might have potential for phytoremediation of metal‐contaminated soils. To further understand the exact degree of Pb accumulated in the plants, BCF and TF were used as an indication of phytoremediation in the study. According to the study of Yoon, Cao, Zhou, and Ma (2006), the BCF value was reported under the category of hyperaccumulators, with a value < 1 denoting metal excluders. In our research, BCF was greater than 1 in *Canna generalis,* while the values of BCF were less than 1 in the other plants (Table 2), indicating that *Canna generalis* is the most suitable plant for use in phytoremediation. TFs were less than 1 in all plants (Table 2), indicating that Pb preferentially accumulated in the external part of the roots and did not translocate to aerial parts, which may be useful information with regard to phyto‐stabilizing Pb traits together with ornamental plants. Similar results have also been also reported by Pal, Banerjee, and Kundu (2013) and Zhao, Xiong, Li, and Zhu (2009), who showed that Pb accumulated mostly in roots, while a small quantity was translocated to shoots. In addition, the lower R/S, which was expressed as the root biomass/aboveground biomass for most plants in Pb‐contaminated soil (Table 1), confirmed the sequestration of Pb in roots and consequent severe inhibition of plant root activities (Figure 2b).

To identify the degree of injury of plant exposed to Pb stress, MTI (expressed by the electrolyte leakage) was determined. MTI, as an indirect measure of plant cell membrane damage, has been widely used to quantify plant cell membrane permeability under environment stress conditions, with a higher membrane thermostability index indicating a loss of membrane permeability (Habiba et al., 2015; Yan et al., 2010). In the present work, the higher Pb levels in soil significantly escalated the leaf MTI (Figure 3c) and enhanced the permeability of membranes, resulting in a loss of membrane integrity in the leaves of all ornamental plants. Similar results have been reported demonstrating that soil Pb increases the conductivity of electrolyte leakage in wheat (Chen, Chen, & Liu, 2017; Kaur, Singh, Batish, & Kohli, 2012), sesame (Mehmood et al., 2018), rice (Ashraf & Tang, 2017), pepper (Kaya, Akram, Sürücü, & Ashraf, 2019), and *Coronopus didymus* (Sidhu, Singh, Batish, & Kohli, 2016). There are two possible explanations for the increase in MTI. One explanation is the generation of ROS, such as hydrogen peroxide, in Pb‐stressed plant leaves caused lipid and protein degradation/oxidation (Dias, Mariz Ponte, & Santos c, 2019). The other one explanation is the binding of the metal to the sulfhydryl group and the destabilization of the membrane by forming disulfide bonds, causing an alteration in the organization and function of membrane ion channels (Aravind & Prasad, 2005). Furthermore, the highest MTI values in response to the same soil Pb treatment were found in the leaves of *Iris germanica* L., indicating that Pb stress posed a higher threat to *Iris germanica* L. than the other ornamental plants.

When suffered environment stress, plants had a self‐protection and stress tolerant ability in avoiding damage caused by ROS. In our study, two parameters, Pro and SS, were determined to identify the physicochemical mechanisms of plant resistance and tolerance to soil Pb stress. The accumulation of Pro and SS stimulated by various metal ions such as Cd^2+^, Pb^2+^, Zn^2+^, Cu^2+^, and Al^3+^ has been widely reported in several studies as one of the most commonly induced adaptive responses of plants (Jia, Zhang, Zhao, Liu, & He, 2018; Li, Yang, Jia, Chen, & Wei, 2013; Nedjimi & Daoud, 2009; Sandra, Marija, Dragan, Vibor, & Branka, 2010; Shevyakova, Netronina, Aronova, & Kuznetsov, 2003), and this effect was greater in shoots than in roots (Tian, Guo, & Yan, 2007; Verma & Dubey, 2001). The accumulation of Pro in the cytosol under heavy metal exposure is not only regarded as an indicator of environmental stress but also to play an important protective role against heavy metal stress (Alia & Saradhi, 1991; Sun, Zhou, Sun, & Jin, 2007) by maintaining the osmotic equilibrium within plant cells (Szabados & Savourcb, 2010), scavenging hydroxyl radicals and singlet oxygen (Kavi & Sreenivasulu, 2014; Matysik, Bhalu, Mohanty, & Bohrweg, 2002; Pal et al., 2013), chelating metal ions in plants and forming a nontoxic metal‐proline complex (Sharma, Schat, & Vooijs, 1998). In our study, the Pro contents in the leaves of *Hemerocallis fulva*, *Iris germanica* L., *Canna generalis*, and *Pennisetum clandestinum* were enhanced consistently with the application of 50, 100, and 200 and 500 mg/kg Pb (Figure 3a). However, at a high concentration of Pb (1,000 mg/kg), excluding *Canna generalis*, the contents of Pro in the leaves of the other three ornamental plants (*Hemerocallis fulva*, *Iris germanica* L., and *Pennisetum clandestinum*) showed a remarkable decline. For *Miscanthus sinensis*, the leaf proline contents increased in response to the 50, 100, and 200 mg/kg Pb treatments but significantly decreased following 500 mg/kg Pb treatment. These results indicated that the application of 500 mg/kg Pb to *Miscanthus sinensis* and 1,000 mg/kg Pb to *Hemerocallis fulva*, *Iris germanica* L., *Pennisetum clandestinum,* and *Miscanthus sinensis* exceeded their abilities to protect themselves against ROS‐induced cell damage (Matysik et al., 2002). The accumulation of SS, which acts as an osmotic agent in stressed plants, has also been considered to be a resistance mechanism to the stress condition (Roitsch, 1999; Wu & Xia, 2006) and to play a pivotal role in the osmotic adjustment in plants (Sánchez, Manzanares, Andres, Tenorio, & Ayerbe, 1998; Zhou & Yu, 2009). Lehner (2008) found that SS could detoxify ROS and was related to ROS‐producing metabolic pathways in wheat. In this study, we found that the contents of SS in leaves of ornamental plants subjected to low levels of Pb (50–200 mg/kg) were significantly higher than in the control group, suggesting that mild Pb stress could enhance the synthesis of carbohydrates to eliminate ROS by reinforcing the antioxidant system and protecting the cells from damage (Nguyen, Hailstones, Wilkes, & Sutton, 2010). Our results are in line with the findings of John, Ahmad, Gadgil, and Sharma (2008), who reported a significant increase in SS contents in *Lemna polyrrhiza* following exposure to low Pb concentration stress. Conversely, high levels (500–1,000 mg/kg) of Pb toxicity reduced soluble sugar concentrations in the leaves of all ornamental plants. The decrease of soluble sugar contents at the high levels of lead in soil might be attributed to the lead‐induced detrimental effect on the structure and function of the photosynthetic apparatus, inhibited the plant carbon assimilation ability, and reduced the synthesis of leaf carbohydrate in the end (Jiang, Wang, Dong, & Yan, 2019). Similar results have also been reported by Bhardwaj, Chaturvedi, and Pratti (2009), Sinha (2004), and Ali et al. (2014), demonstrating the dose dependence of the soluble sugar concentration in ornamental plants exposed to Pb treatment.

Photosynthetic abilities of plants have been shown to be vulnerable to heavy metals (Boucher & Carpentier, 1999; Tanyolaç, Ekmekçi, & Ünalan, 2007). Several authors have reported negative effects on photosynthetic capacity in different species grown in media with high concentrations of Pb (Briat & Lebrun, 1999; Ferreyroa, Lagorio, Trinelli, Lavado, & Molina, 2017; Legocka, Sobieszczuk‐Nowicka, Wojtyla, & Samardakiewicz, 2015; Yang et al., 2015; Zhou, Jiang, Ma, Yang, & Wei, 2017). Our study provided similar results showing that soil Pb even at low doses, and despite the small amount of translocation from root s to shoots (validated by TF < 1, Table 2), inhibited the photosynthetic rate in the leaves of all ornamental plants (Figure 2a). The inhibition mechanisms of Pb stress on plant carbon assimilation ability are associated with the destruction of the chloroplast ultrastructure, alteration of the photosynthetic apparatus functionality, and reduction of antioxidant catalase activity, among others (Arena et al., 2017; Islam et al., 2008; Tian et al., 2014). However, the primary restriction factor for plant photosynthetic capacity under Pb stress conditions was species‐specific (Bouchereau, Aziz, Larher, & Martin‐Tanguy, 1999; Sánchez et al., 2001; Wang & Rainbow, 2006; Yang et al., 2011). In the present study, methods of all‐subsets regression and linear regression were used to elucidate the relationship of *P*_n_ with the Pro, SS, MSI, and Chl contents in the leaves of different ornamental plants. According to the linear relationship between *P*_n_ and other parameters, there was remarkable evidence that plant photosynthesis had a significant positive relationship with the leaf Chl content, especially Chl *b*, for all ornamental plants (Table 5). These results indicated that chloroplasts might be the primary target for heavy metal toxicity in terms of the plant photosynthetic capacity of ornamental plants, which is consistent with the findings of a study on *Chlorella kessleri* (Sabatini et al., 2009). In fact, Chl content has been widely demonstrated to play a crucial role and act as an important indicator of plant photosynthetic potential under heavy metal stress conditions (Piotrowska‐Niczyporuk, Bajguz, Zambrzycka, & Godlewska‐Żyłkiewicz, 2012). Many mechanisms have been developed to explore the decrease in Chl content caused by Pb stress. According to the study of Mishra et al. (2006), the inhibition of photosynthetic pigment synthesis, disruption of the uptake of essential ions such as Mg^2+^, Fe^2+^, and Mn^2+^, and increase in chlorophyllase activity catalyzing chlorophyll degradation resulted in the reduction of Chl. The other explanation for the decrease in Chl content was that the heavy metal ion substitutes for Mg^2+^ in the tetrapyrrole ring of the chlorophyll molecule, destroying the chloroplast apparatus (Çelekli, Kapı, & Bozkurt, 2013). Although the reduction of chlorophyll *a* + *b* content had been widely detected in leaves of plants, such as *Vallisneria natans* (Wang, Zhang, Wang, & Lu, 2012), *Ceratophyllum demersum* L. (Mishra et al., 2006), and *Elodea canadensis* (Dogan, Saygideger, & Colak, 2009), the susceptibilities of chloroplast a and b were different in different plant species. Gajewska, Skłodowska, Słaba, and Mazur (2006) and Xiong, Zhao, and Li (2010) revealed a more significant decline in Chl *b* than Chl *a* under heavy metal stress, while Hu et al. (2012) reports a contradictory result. Our results are consistent with former studies showing a more sensitive response of Chl *b* than Chl *a* in the leaves of ornamental plants exposed to Pb stress. The results showed that most soil Pb treatments had no significant effect on the Chl *a* concentration (Figure 1a) in leaves of *Hemerocallis fulva*, *Iris germanica* L., *Pennisetum clandestinum*, and *Miscanthus sinensis*, but the Chl *b* concentration was markedly reduced in most ornamental plants (excluding *Iris germanica* L.), even at a low Pb concentration (50–100 Pb mg/kg; Figure 1b), causing a significantly lower total pigment concentration in Pb‐treated plants than the control (Figure 1c).

**TABLE 5:** Linear relationship of the net photosynthetic rate with chlorophyll and soluble sugar contents in leaves of *Hemerocallis fulva*, *Iris germanica* L., *Canna generalis*, *Pennisetum clandestinum*, and *Miscanthus sinensis*

Plant species	Linear equation	*R* ^2^	*p*
*Hemerocallis fulva*	*P*_n_ = −21.7 – 4.1 × Chl *b*	.82	<.01
*Iris germanica* L.	*P*_n_ = −1.1 + 1.2 × Chl *a* + 1.2 × Chl *b*	.75	<.01
*Canna generalis*	*P*_n_ = 0.3 + 3.4 × Chl *b*	.67	<.01
*Pennisetum clandestinum*	*P*_n_ = 22.7 + 23.8 × Chl *b*	.83	<.01
*Miscanthus sinensis*	*P*_n_ = 8.3 + 1.1 × Chl *b* + 0.0025 × SS	.91	<.01

The identification of tolerant native plant species is essential for the phytoremediation of heavy metal‐contaminated soils (Asgari Lajayer et al., 2017). The evaluation of Pb tolerance was complicated and revealed species specificity in different plants. Although numerous parameters, such as the catalytic activities of antioxidant enzymes (superoxide dismutase, ascorbate peroxidase, and guaiacol peroxidase) in *Vallisneria natans* (Wang et al., 2011), K^+^/Na^+^ ratio in *Helianthus annuus* L. (Hao, Zhou, Li, & Jiang, 2012), growth tolerance index in *Dianthus carthusianorum* L. (Muszynska et al., 2018), leaf malondialdehyde level in *Pogonatherum crinitum* seedlings (Hou et al., 2018), plant growth rates in *Nerium oleander* L. (Trigueros, Mingorance, & Rossini Oliva, 2012), organic acid in shoots of *Echium vulgare* L. (Dresler et al., 2017), were used to distinguish plant Pb tolerance, the use of a single or a few evaluation indicators may not comprehensively reflect the abilities of plants to adapt to Pb stress conditions. To solve this limitation, in our research, CEV, which is based on the combination of cluster analysis and the standard deviation coefficient allocation weighted method, was used to provide a comprehensive evaluation of the Pb tolerance of five ornamental plants. Based on the comprehensive evaluation value for each ornamental plant species shown in Table 4, *Iris germanica* L. had the lowest value among all Pb treatments, suggesting that *Iris germanica* L. had the weakest ability to tolerate Pb stress. In contrast, *Canna generalis* and *Pennisetum clandestinum* had a higher value in response to all Pb treatments compared with the other ornamental plants, indicating that they were the two most tolerant species to Pb stress.

Cluster and heatmap analyses as useful tools were used in the present study for measurement data reduction (Gu et al., 2012). Agglomerative hierarchical cluster analysis was applied, in which 29 variables (Pb‐treated plant species) were selected and a two‐dimensional visualization technique (heatmap) based on the Spearman correlation matrix of the 17 variables (plant physiological and biochemical parameters) was generated, as shown in Figure 4, to select key variables for different plant species. In this figure, weak correlations between variables are displayed in green and yellow, while stronger correlations are shown in red. The cluster analysis classified different Pb‐treated plant species into five subtypes, which were believed to best represent each cluster. Interestingly, the five classified subtypes presented a very close homologies with plant genotypes under different soil Pb conditions, indicating that different ornamental plant species presented unique physiological and biochemical responses to soil Pb stress. Based on the heatmap, a key variable was selected for each plant. According to the red colour in the heatmap, the key parameter in the responses to different soil Pb treatments was plant height for *Hemerocallis fulva*, Chl *a*/*b* for *Iris germanica* L., soluble sugar for *Canna generalis*, Chl *b* for *Pennisetum clandestinum,* and root activity for *Miscanthus sinensis*.

## 5 CONCLUSIONS

**FIGURE 5:**
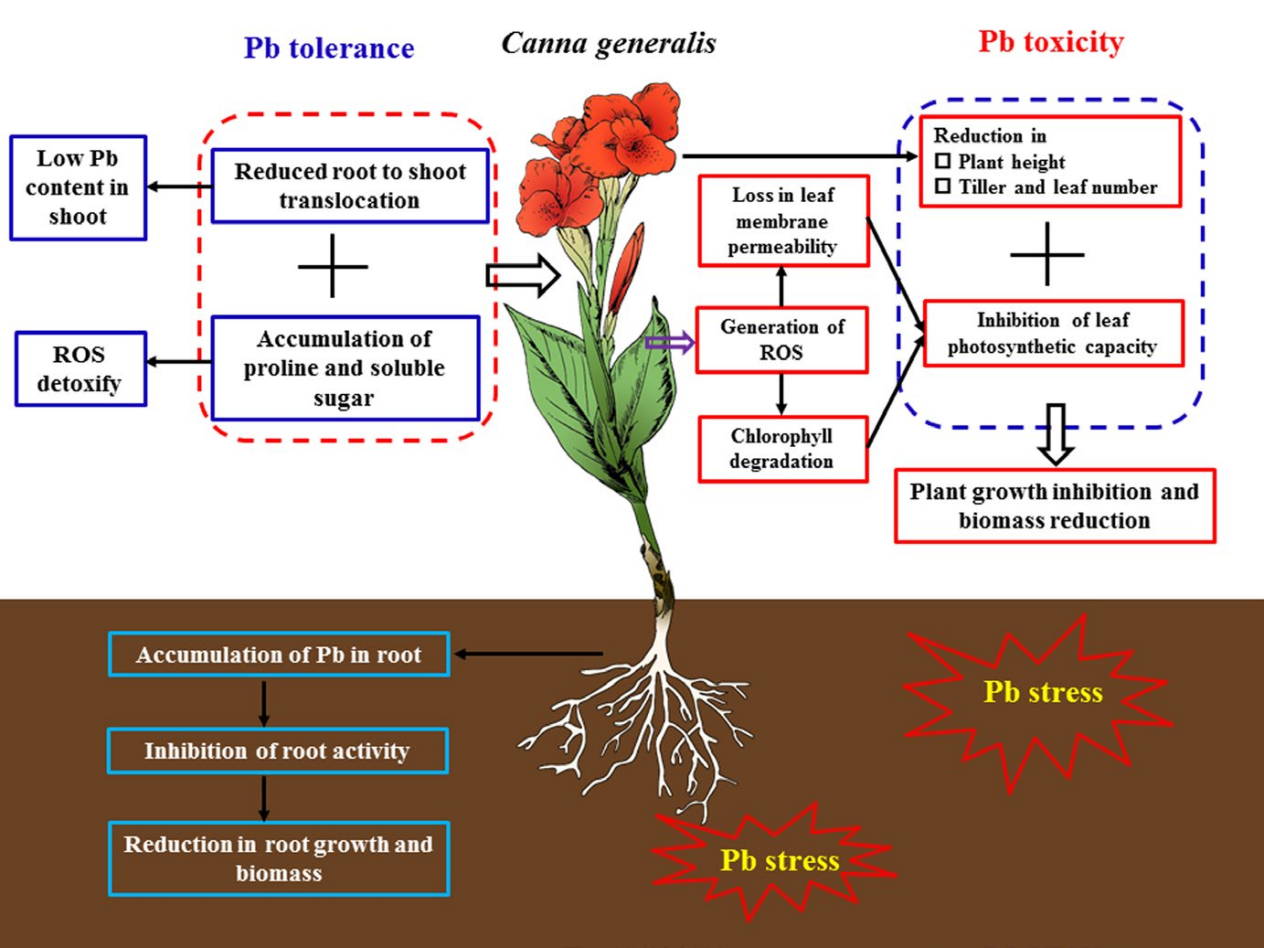
Schematic representation of the Pb toxicity on the shoot and root of ornamental plants and the plant tolerance mechanisms under Pb stress conditions

In this study, several physiological and biochemical parameters were determined and analyzed along with the Pb uptake and tolerance abilities in *Hemerocallis fulva*, *Iris germanica* L., *Canna generalis*, *Pennisetum clandestinum,* and *Miscanthus sinensis,* with the purpose of testing the negative effect of Pb stress on ornamental plants, exploring plant tolerance mechanisms in Pb toxicity, and elucidating the most suitable plant for use in phytoremediation. Figure 5 shows the Pb toxicity towards the shoots and roots of ornamental plants and their tolerance mechanisms under conditions of Pb stress. Although Pb ion mainly accumulated in plant roots and the aerial parts of plants had a relatively low Pb content, six weeks of soil Pb treatments caused acute Pb toxicity in both shoots and roots. Pb stress decreased all plant growth traits (e.g., plant height, leaf area, leaf number, and tiller number), reduced root activity, enhanced leaf membrane permeability, and inhibited plant photosynthetic capacity by reducing the leaf Chl content, resulting in a significant reduction of plant growth and biomass in all the ornamental plants. Proline and soluble sugars were also accumulated in the Pb‐stressed leaves of ornamental plants in all Pb treatments, but their self‐protective abilities against Pb stress were downregulated at high Pb levels (500–1,000 mg/kg). By calculating the CEV, *Iris germanica* L. was found to be the most sensitive plant to Pb stress, and *Canna generalis* was the most suitable plant for use in phytoremediation.

## CONFLICT OF INTEREST

The authors declare no conflict of interest.

[Correction added on 24 May 2021, after first online publication: Conflict of Interest statement added to provide full transparency.]

## AUTHOR CONTRIBUTIONS

WF Chen and CX Zhang conceived and conducted the experiment. XL Song, YH Zhu, and YY Wang analyzed the results. and XL Song wrote the article. All authors reviewed the manuscript.

## Data Availability

The data that support the findings of this study are available from the corresponding author upon reasonable request.
